# Evaluation of the effects of the recommended oral dose of diflubenzuron on bovine sperm and oocyte quality using CASA and OPU-IVEP

**DOI:** 10.3389/fvets.2023.1215722

**Published:** 2023-07-11

**Authors:** Marcelo Cunha Xavier, Leticia Prates Martins, Rodrigo Martins Moura, Divino Fabio Morais, Joao Vitor Lima Barbosa, Ricardo Alamino Figueiredo, Mauricio Antonio Silva Peixer, Rosangela Vieira de Andrade, Joao Henrique Moreira Viana

**Affiliations:** ^1^Programa de Pós-Graduação em Ciências Genômicas e Biotecnologia, Universidade Católica de Brasília, Brasília, DF, Brazil; ^2^Bio Biotecnologia da Reprodução Animal, Brasília, DF, Brazil; ^3^Programa de Pós-Graduação em Biologia Animal, Universidade de Brasília, Brasília, DF, Brazil; ^4^Departamento de Medicina Veterinária, Centro Universitário de Brasília, Brasília, DF, Brazil; ^5^Embrapa Recursos Genéticos e Biotecnologia, Brasília, DF, Brazil

**Keywords:** Difly, reproductive disruptors, *in vitro* embryo production, semen, Nelore breed

## Abstract

**Introduction:**

The aim of this study was to evaluate potential effects of diflubenzuron on the production and quality of gametes, and on *in vitro* embryo production (IVEP) outcomes, in cattle.

**Methods:**

Two experiments were performed, the first to evaluate effects on semen, and the second on *cumulus*-oocyte complexes (COC) and on IVEP. Nelore (*Bos taurus indicus*) bulls (*n* = 14) or heifers (*n* = 16) were allocated into control (CG) or treatment (DIF) groups. All groups received a mineral mix supplement added (DIF) or not (CG) with diflubenzuron (30 mg/head/day), during 8 weeks. Animals were weighed and blood samples were collected throughout the experimental period. Every other week, bulls were subjected to semen collection and heifers to transvaginal ultrasound-guided follicle aspiration sessions. Semen underwent physical and morphological evaluation, and samples were stored for further computer-assisted sperm analysis. The COC recovered were evaluated according to morphology and those classified as viable were sent to an IVEP laboratory.

**Results:**

Diflubenzuron had no effect (*P* > 0.05) on average body weight or in any blood hematological or biochemical endpoints, regardless of gender. In experiment 1, there was no difference (*P* > 0.05) between DIF and CG groups for sperm concentration, morphology, or kinetics. In experiment 2, there was also no effect of diflubenzuron on the number of total, viable, or grade I oocytes, as well as on cleavage or blastocyst rates (*P* > 0.05).

**Discussion:**

In summary, the oral administration of diflubenzuron, within the recommended dose, has no short-term negative effects on sperm production and quality or on oocyte yield and developmental potential *in vitro*, in cattle.

## 1. Introduction

The diflubenzuron [1-(4-clorophenyl)-3-(2,6-difluorobenzoyl) urea] is a chemical from the benzoylphenyl urea (BPU) class widely used since the 1970′s for pest control, both in urban areas and in agriculture and livestock (1). It inhibits synthesis of chitin exoskeleton and acts as an arthropod larvae growth regulator, being used as an insecticide and acaricide. In cattle, diflubenzuron has been used as a diet additive to control horn fly (*Haematobia irritans*), housefly (*Musca domestica*), stablefly (*Stomoxys calcitrans*), as well as ticks *(Rhipicephalus microplus)*. Diflubenzuron is poorly absorbed by the gastrointestinal track of cattle, extensively metabolized, and almost totally excreted in feces and urine, resulting in very low residue concentrations in milk and meat (2, 3). Within the recommended doses, such residues are usually undetectable or below the acceptable daily intake (ADI) values (4), and thus are unlikely to present a public health concern (1, 5). Therefore, there is no withdraw time, and diflubenzuron can be used in lactating dairy cattle (6).

Although diflubenzuron has been considered as safe for use in livestock, results from toxicological studies on non-target species are controversial. No carcinogenic effect of diflubenzuron was observed *in vitro* (7, 8), whereas dose-dependent genotoxic and mutagenic effects were observed *in vivo* in some animal models (9), but not others (10). Such inconsistencies may be associated with the indirect effect of metabolites, such as 4-chloroaniline, which are formed *in vivo* from the small proportion of the diflubenzuron that is actually absorbed (11). Recently, diflubenzuron was shown to induce cell apoptosis and ROS generation in mammary epithelial cells (12). Potentially detrimental effects for cell and especially for DNA integrity are particularly important for gametes and embryos, and thus for reproductive toxicity. It is also noteworthy that diflubenzuron, besides acting on chitin synthesis on larvae, also impairs oogenesis and reduces ovarian follicle population in adult insects (13). In this regard, potential actions on gametogenesis in other species should be a target for risk assessment. Nevertheless, very few studies have addressed the potential reproductive toxicity of diflubenzuron on livestock.

*In vitro* embryo production (IVEP) has become the technique of choice for embryo production in many countries, and in 2020 the number of embryos produced *in vitro* was three times greater than the number of embryos collected *in vivo* worldwide (14). Besides its key role as a tool for animal breeding programs, IVEP also provides an important platform for the study of environmental effects, including endocrine disruptors, on reproduction. *In vitro* maturation (IVM) of bovine oocytes, for example, has been proposed as a potential method to evaluate chemical hazards on fertility, requiring far fewer animals to demonstrate potential detrimental effects on reproduction than *in vivo*-models (15). The bovine species is also a suitable model for human reproductive toxicology studies, due to the similarities between bovine and human ovarian physiology (16). Similarly, the advances in bovine semen evaluation, such as the use of computer-assisted sperm analysis (CASA), has provided more accurate tools to identify potential interferences in male reproductive physiology, e.g., minor changes in sperm quality not detectable in routine andrological exams.

The aim of the present study was to evaluate possible short-term potential detrimental effects of diflubenzuron treatment on gamete quantity, quality, and developmental potential in cattle. Our hypothesis was that, within the current recommended doses, diflubenzuron treatment would not affect gamete or embryo quality and developmental potential in cattle. The rationale for this study was that the use of new analytical methods, such as CASA, and *in vivo*-*in vitro* experimental models such as the association of *in vivo* oocyte recovery with IVEP, are important to validate previous toxicological evaluations on the safety of diflubenzuron on livestock.

## 2. Material and methods

### 2.1. Animals and location

Experiment 1 was conducted on a private beef farm located in Formosa, GO, Brazil. Pubertal Nelore (*Bos taurus indicus*) bulls (*n* = 14), with no detectable abnormalities during andrological exams, were enrolled. The bulls were kept under Brachiaria sp. pasture with ad libitum access to water, and received 2 Kg/head/day of concentrate supplement with a mineral mix. Experiment 2 was conducted at the Embrapa's Sucupira Experimental Station, in Brasilia, DF, Brazil. Nulliparous, pubertal, cycling Nelore (*Bos taurus indicus*) heifers (*n* = 16), with no detected pathologies during gynecological examination, were enrolled. The heifers were confined and received a maintenance diet consisting of corn silage and mineral mix (100 g/day/head), starting at an adaptation period 2 weeks before the beginning of the experiment. Both experiments were conducted from June to August (winter, dry season).

### 2.2. Experimental design

**Figure 1:**
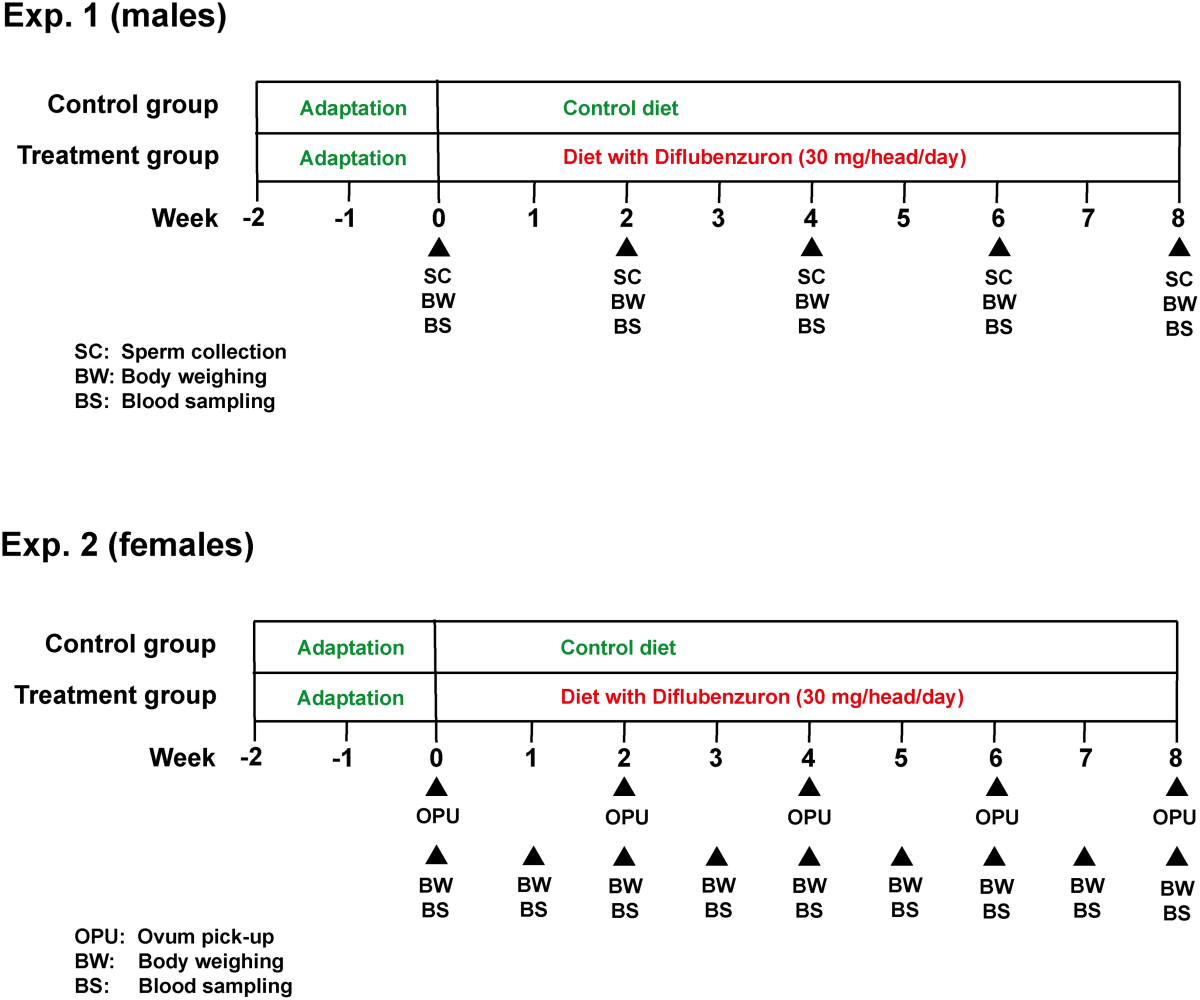
Experimental design used in experiments 1 (males) and 2 (females).

This study was subdivided into two experiments (for bulls and heifers, respectively), both following a similar experimental design (Figure 1). In both experiments, animals were distributed into a control (CG) or treatment (DIF) group. Bulls were allocated to the experimental groups at random, whereas heifers were first ranked according to antral follicle count (AFC), as defined elsewhere (17) and, from the higher to the lower position, alternately allocated to the CG or DIF groups. The balancing of distribution was confirmed by the lack of difference (*P* > 0.05) in AFC between the two groups at the beginning of the experiment. The experimental groups received a mineral mix added (DIF) or not (CG) with diflubenzuron, calculated to meet the daily mineral requirements and, in the DIF groups, to ensure the daily consumption of 1 g of a commercial formulation (Difly S3, Champion, Anápolis, Brazil) with 3% diflubenzuron (30 mg per head/day, according to product label recommendation, https://www.champion.ind.br/produto/difly-s3-6-kg-2/), during 8 weeks (56 days). The consumption of the mineral mix of each group was monitored daily, the confirm the ingestion of the expected diflubenzuron dose. Body weight was evaluated and blood samples were collected weekly in the heifers, and every 15 days in the bulls. Starting immediately before treatments, every other week bulls were subjected to semen collection, and heifers to transvaginal ultrasound-guided follicle aspiration (OPU), in a total of five sessions in each experiment. Semen underwent physical and morphological evaluation, and samples were stored for further computer-assisted sperm analysis (CASA). The *cumulus*-oocyte complexes (COC) recovered were evaluated according to morphology and sent to a commercial laboratory for IVEP.

### 2.3. Blood sampling and blood analyses

Blood samples were collected by puncture of the coccygeal vein or artery, using 21G double-ended needles. Two samples were collected from each animal into 5 mL vacuum tubes, one with and another without K3 EDTA (Vacutainer Systems; Becton Dickinson, São Paulo, SP, Brazil). Samples were kept under refrigeration (5°C) and transported to a commercial veterinary clinical analysis laboratory. Each sample was evaluated for hemogram and biochemical endpoints.

### 2.4. Semen collection and analysis

Semen samples were collected by electroejaculation, as described elsewhere (18). Briefly, bulls were contained in a beef cattle squeeze chute, and ejaculation was induced using an electroejaculator, at presetting #4 (Autojac 3, Neovet, Uberaba, Brazil). Samples (20 μL) of fresh semen were evaluated under x200 magnification for mass movement, vigor, and motility, and under x1000 magnification for morphological analysis of the sperm cells, as previously described (19). The semen was them diluted 1:1 in a semen extender (Optixcell, IMV Technologies Brasil, Campinas, Brazil) and kept under refrigeration (5°C) until transportation to the laboratory. Fresh semen from each bull was rediluted 1:10 in Optixcell and a 3 μL sample was analyzed in a counting chamber (Leja, IMV Technologies) using CASA (SpermVision Minitube GMBH, Tiefenbach, Germany), with the standard presetting of the equipment. The remaining semen was then diluted, according to the sperm concentration determined by CASA, to a final concentration of 50 x 10^6^ sperm/mL, filled into 0.5 mL straws, stabilized at 4°C for 4 h, and frozen using a freezing machine (Cryogen, Neovet) with the standard freezing curve for cattle. Samples of frozen semen were also analyzed by CASA, immediately after thawing or after being submitted to a thermo-resistance test (TRT) during 4 h.

### 2.5. Oocyte recovery and *in vitro* embryo production

All ultrasonographic exams and OPU procedures were performed using a portable ultrasound device (MyLab 30 Gold Vet, Esaote, Genova, Italy) equipped with a 7.5 MHz linear rectal probe or a micro-convex vaginal 7.5 MHz probe mounted in a custom-made polyethylene needle guide (WTA Tecnologia Aplicada, Cravinhos, Brazil). Before the first OPU, the number of sonographic-detectable follicles (AFC) in the ovaries was recorded and used to balance the distribution of heifers between treatments.

The OPU-IVEP procedures used were the same as described elsewhere (20). Briefly, immature COC were collected by puncture and aspiration of visible follicles (>2 mm), using 20 G needles and a vacuum pressure of approximately 80 mm/Hg. The aspirated fluid with the follicle content was recovered into 50 mL tubes containing DPBS added with 1% FCS and 125 IU/mL sodium heparin. COC quality was evaluated under a stereomicroscope at x40 magnification and those morphologically classified as viable, as previously described (21), were transferred to 1.2 mL cryotubes (Corning, New York, USA) containing maturation medium and transported in a portable incubator (Minitub do Brasil, Porto Alegre, Brazil) at 38°C to the IVEP laboratory.

The COC from each donor underwent *in vitro* maturation (IVM), *in vitro* fertilization (IVF), and subsequent *in vitro* embryo culture (IVC) separately. IVM was performed for 20 h in TCM199 (Gibco, New York, USA) supplemented with 0.05 IU/mL FSH (Pluset, Hertape-Calier, Barcelona, Spain), 1 mg/mL estradiol, and 10% FCS in a humidified incubator at 5% CO_2_ in air and 38.5°C. Expanded COC were then partially denuded and transferred to drops with Tyrode's albumin lactate pyruvate (TALP) medium supplemented with 10 μg/mL heparin, 20 μM D-penicillamine, 10 μM hypotaurine, and 1 μM epinephrine. IVF was performed with 1 x 10^6^ spermatozoa/mL for 18 h. Semen from a single Nelore sire with known fertility on IVEP was used for all fertilization batches. Presumptive zygotes were then IVC in 50 μL droplets of synthetic oviduct fluid (SOFaa) supplemented with essential and non-essential amino acids, 0.34 mM sodium tricitrate, 2.77 mM myo-inositol, and 10% FCS, under mineral oil. Cleavage and blastocyst rates were determined at 72 and 168 h post-insemination. Blastocysts were also classified according to developmental stage (initial blastocysts, blastocysts, expanded blastocysts, or hatched blastocysts). A subset of the expanded blastocysts (circa 30/batch/group) were transferred to glass slides, stained with Hoechst 33342 (Sigma-Aldrich, St. Louis, MO, USA), and evaluated under the microscope (x1000) for total cell number (ICM plus trophoblast).

### 2.6. Data analysis

All endpoints related to weight, blood hematological and biochemical analyses, sperm production and quality in the bulls, and oocyte yield and *in vitro* embryo production in heifers, were compared between CG and DIF groups using the GLIMMIX procedure of SAS (SAS Studio 3.8, University Edition; SAS Institute Inc., Cary, NC, USA) with a repeated statement to account for measurements over time. The model included the effects of treatment (control or diflubenzuron), time (semen collection or OPU-IVEP session), and their interactions, and was adjusted for the type of distribution of each variable. Due to low individual frequencies, sperm pathologies were pooled as major or minor defects, as described elsewhere (22). Similarly, expanded blastocysts were grouped according to cell count in those with >100 or < 100 cells. Differences among means were determined using the Student *T*-test. Results are shown as mean ± SEM. Exact *P*-values are shown for each comparison, to denote how close mean differences were from significance (*P* < 0.05).

## 3. Results

The average body weight and blood parameters in Nelore bulls and heifers from the CG and DIF groups are shown in Table 1. In general, there was no effect (*P* > 0.05) of treatment on any of the endpoints analyzed for both males and females. A time-effect (*P* > 0.05) was observed on weight in males and on most blood parameters in both groups and gender, but a treatment x time interaction was only observed for creatinine and alkaline phosphatase (ALP) in males.

**Table 1:** Body weight and blood hematological and biochemical endpoints (mean ± SEM) of Nelore (*Bos taurus indicus*) bulls and heifers treated (DIF) or not (CG) with 30 mg diflubenzuron/day, during 8 weeks.

**Endpoint**	**Gender^a^**	**CG**	**DIF**	***P*** **-value**			**Ref. value^b^**
				**Treatment**	**Time**	**Treatment × time**	
Weight (Kg)	M	728.9 ± 20.3	715.5 ± 24.1	0.9831	0.0123	0.2163	–
	F	524.1 ± 5.3	559.5 ± 7.9	0.1057	0.7229	0.9995	
Hematocrit (%)	M	39.0 ± 0.8	41.1 ± 0.7	0.2156	0.4779	0.9466	24.0–46.0
	F	42.8 ± 0.5	40.9 ± 0.5	0.1842	0.0023	0.1386	
Protein (g/dL)	M	7.7 ± 0.1	7.9 ± 0.1	0.1487	0.0462	0.6179	7.0–8.5
	F	7.2 ± 0.1	7.2 ± 0.1	0.5855	< 0.0001	0.0982	
Creatinine (mg/dL)	M	2.5 ± 0.0	2.3 ± 0.1	0.3294	0.0050	0.0419	1.0–2.0
	F	2.5 ± 0.2	2.6 ± 0.1	0.2274	0.0074	0.6820	
ALP^c^ (U/L)	M	191.9 ± 10.0	185.7 ± 10.6	0.7244	0.0034	0.0003	90–170
	F	124.6 ± 3.7	120.4 ± 3.1	0.7806	0.0395	0.5575	

^a^M, males; F, females.

^b^Physiological reference values for cattle adopted by the Laboratory in charge of the analyses.

^c^Alkaline phosphatase.

### 3.1. Experiment 1

Two bulls did not adapt to the semen collection routine and were removed from the experiment, therefore the CG and DIF groups consisted of five and seven bulls, respectively. The treatment with diflubenzuron had no effect (*P* > 0.05) on any physical or CASA sperm parameter, regardless of whether the analyses were carried out with fresh, frozen-thawed, or frozen-thawed plus TRT semen (Table 2). Conversely, there was a time-effect (*P* < 0.05) on most endpoints. Treatment x time effects were seldom observed, in all cases associated with fluctuations in values, rather than with increasing or decreasing trends throughout the experimental period.

**Table 2:** Sperm endpoints (mean ± SEM) in Nelore (*Bos taurus indicus*) bulls treated (DIF, *n* = 7) or not (CG, *n* = 5) with 30 mg diflubenzuron/day, during 8 weeks.

**Endpoint**	**CG**	**DIF**	***P*** **-value**		
			**Treatment**	**Time**	**Treatment × time**
**Physical exam**					
Volume (mL)	6.6 ± 0.4	4.4 ± 0.5	0.0634	0.2365	0.0250
Motility (%)	65.6 ± 2.6	65.5 ± 4.0	0.4239	0.6457	0.1908
Vigor^a^	3.6 ± 0.1	3.6 ± 0.2	0.4205	0.0053	0.0131
Mass movement^a^	2.5 ± 0.3	2.7 ± 0.3	0.6363	0.0001	0.4567
Major defects (%)^b^	21.5 ± 2.0	14.1 ± 1.3	0.0569	0.4750	0.2126
Minor defects (%)^c^	8.3 ± 1.9	12.6 ± 2.5	0.2866	0.0356	0.3536
**CASA parameters** ^d^					
**Fresh semen**					
MOT (%)	81.8 ± 2.3	76.4 ± 3.5	0.6410	0.7706	0.7290
DCL (μm)	80.3 ± 2.1	81.5 ± 3.1	0.6125	0.0032	0.0505
DAP (μm)	39.1 ± 0.9	40.1 ± 1.4	0.3150	0.022	0.0526
DSL (μm)	23.1 ± 0.4	24.5 ± 0.9	0.0690	0.0087	0.0407
VCL (μm/s)	185.3 ± 5.0	187.2 ± 7.0	0.6252	0.0047	0.1637
VAP (μm/s)	90.6 ± 2.1	92.5 ± 3.3	0.3205	0.0258	0.1908
VSL (μm/s)	53.8 ± 1.1	57.1 ± 2.0	0.0632	0.0078	0.1105
LIN (%)	0.3 ± 0.0	0.3 ± 0.0	0.1773	0.0001	0.7351
STR (%)	0.6 ± 0.0	0.6 ± 0.0	0.2532	0.0001	0.8774
WOB (%)	0.5 ± 0.0	0.5 ± 0.0	0.3189	0.0001	0.1447
BCF (Hz)	24.6 ± 0.4	24.6 ± 0.8	0.2960	0.2244	0.1429
ALH (μm)	7.3 ± 0.2	7.1 ± 0.3	0.8133	0.1458	0.6122
**Frozen/thawed semen**					
MOT (%)	28.3 ± 2.9	27.8 ± 2.7	0.8516	0.0001	0.0132
DCL (μm)	60.7 ± 2.4	63.1 ± 3.2	0.6801	0.0033	0.1851
DAP (μm)	30.8 ± 0.9	32.8 ± 1.3	0.4527	0.0081	0.1210
DSL (μm)	22.5 ± 0.6	24.3 ± 0.9	0.3547	0.0032	0.0281
VCL (μm/s)	136.6 ± 5.0	143.1 ± 6.8	0.5954	0.0032	0.1158
VAP (μm/s)	69.8 ± 1.8	74.9 ± 2.7	0.3450	0.0096	0.0618
VSL (μm/s)	50.9 ± 1.3	55.7 ± 1.7	0.2152	0.0057	0.0104
LIN (%)	0.4 ± 0.0	0.4 ± 0.0	0.2948	0.0024	0.8670
STR (%)	0.7 ± 0.0	0.7 ± 0.0	0.5747	0.4309	0.5898
WOB (%)	0.5 ± 0.0	0.5 ± 0.0	0.2144	0.0001	0.8608
BCF (Hz)	25.3 ± 0.8	25.1 ± 0.7	0.8463	0.0628	0.3057
ALH (μm)	5.4 ± 0.1	5.5 ± 0.1	0.7235	0.3370	0.0077
**Thawed semen after TRT** ^e^					
MOT (%)	21.5 ± 2.5	18.8 ± 2.0	0.2467	0.0001	0.0097
DCL (μm)	48.6 ± 2.6	41.1 ± 1.9	0.1054	0.0029	0.5368
DAP (μm)	26.5 ± 1.1	23.3 ± 0.8	0.0787	0.0009	0.4765
DSL (μm)	20.7 ± 0.8	18.4 ± 0.6	0.0639	0.0013	0.4316
VCL (μm/s)	108.5 ± 5.6	92.3 ± 3.8	0.0935	0.0031	0.3612
VAP (μm/s)	59.6 ± 2.5	53.1 ± 1.5	0.0711	0.0003	0.2227
VSL (μm/s)	46.6 ± 1.8	42.2 ± 1.1	0.0584	0.0003	0.2101
LIN (%)	0.4 ± 0.0	0.5 ± 0.0	0.2065	0.0153	0.9822
STR (%)	0.8 ± 0.0	0.8 ± 0.0	0.4490	0.0388	0.6685
WOB (%)	0.6 ± 0.0	0.6 ± 0.0	0.1695	0.0141	0.9821
BCF (Hz)	25.0 ± 0.8	22.2 ± 0.8	0.0682	0.0516	0.5585
ALH (μm)	4.2 ± 0.2	4.2 ± 0.1	0.7757	0.1680	0.0117

^a^Subjective score (1–5).

^b^Major defects: defective acrosome; amorphous head; pouch formation; head narrow at the base; detached abnormal head; microcephalic head; pyriform head; bent tail with distal cytoplasmic droplet; coiled tail; folded tail; corkscrew defect; teratoid forms; proximal cytoplasmic droplet; midpiece defects.

^c^Minor defects: pseudo-droplets; detached acrosome; narrow head; giant head; detached head; twisted tail; bent tail; distal cytoplasmic droplet; abaxial tail implantation.

^d^MOT, Motility; DCL, Curvilinear Distance; DAP, Distance of Average Path; DSL, Straight Line Distance; VCL, curvilinear velocity; VAP, average path velocity; VSL, continuous line velocity; LIN, linearity; STR, straightness; WOB, Wobble coefficient; BCF, beat cross frequency; ALH, amplitude of lateral head displacement.

^e^Thermo-resistance test.

### 3.2. Experiment 2

Data related to oocyte recovery and *in vitro* embryo production are shown in Table 3. During the five OPU sessions, the CG and DIF groups yielded 801 and 986 total COC, and 631 and 797 viable COC, respectively. The third IVEP session was affected by microbiological contamination in both groups during IVF, thus data from this IVEP session was discarded. Similarly to what was observed for semen, a time-effect was observed for most OPU-IVEP endpoints, whereas there was no effect (*P* > 0.05) of the diflubenzuron treatment on any parameter. A treatment x time interaction was observed in the number of follicles aspirated, which decreased (*P* < 0.05) consistently in CG but increased (*P* < 0.05) from sessions three to four in DIF group, and in the proportion of expanded blastocysts and of embryos with > 100 cells, which fluctuated in both groups.

**Table 3:** OPU-IVEP outcomes (mean ± SEM) in Nelore (*Bos taurus indicus*) heifers treated (DIF) or not (CG) with 30 mg diflubenzuron/day, during 8 weeks.

**Endpoint**	**CG**	**DIF**	***P*** **-value**		
			**Treatment**	**Time**	**Treatment × time**
OPUs (*n*)	40	40			
Follicles aspirated (*n*)	27.4 ± 2.1	26.7 ± 1.7	0.8826	0.0403	0.0114
Total COC (*n*)	20.0 ± 1.8	24.7 ± 1.9	0.3694	0.0320	0.1423
Recovery rate (%)	78.1 ± 3.1	88.4 ± 2.4	0.0957	0.1517	0.1786
Viable COC (*n*)	15.8 ± 1.6	19.9 ± 1.8	0.3947	0.5842	0.2358
Viable rate (%)	77.9 ± 2.0	78.7 ± 1.9	0.8202	< 0.0001	0.8760
COC Grade I (*n*)	2.4 ± 0.4	3.4 ± 0.6	0.3242	0.0005	0.1747
Denuded oocyte (*n*)	0.4 ± 0.1	0.8 ± 0.2	0.1213	0.0286	0.8598
Degenerated COC (*n*)	2.0 ± 0.3	2.3 ± 0.2	0.4203	0.0010	0.9885
Expanded COC (*n*)	2.4 ± 0.3	2.1 ± 0.3	0.6497	< 0.0001	0.1420
IVEP (*n*)^a^	32	32			
Cleaved (*n*)	9.2 ± 1.3	14.5 ± 2.0	0.1913	0.1373	0.7390
Cleavage rate (%)	58.9 ± 3.8	67.5 ± 3.3	0.2893	0.0025	0.7041
Blastocysts (*n*)	6.1 ± 0.9	9.6 ± 1.2	0.1873	0.0781	0.8595
Blastocyst rate (%)	39.7 ± 3.5	44.5 ± 2.4	0.5301	0.0127	0.2719
Expanded blastocysts (*n*)	4.0 ± 0.7	6.2 ± 0.9	0.2743	0.2368	0.7597
Expanded blastocysts (%)^b^	64.2 ± 5.7	63.3 ± 4.1	0.7913	0.1449	0.0127
Embryos > 100 cells (*n*)	3.5 ± 0.6	3.7 ± 0.6	0.9452	0.0450	0.0010

^a^One IVEP session was lost due to contamination and excluded from calculations.

^b^Calculated over the total of blastocysts.

## 4. Discussion

Benzoylurea chitin inhibitors, such as diflubenzuron, are selective pesticides with known detrimental effects on arthropods, as wells as in other organisms presenting chitin in their shells or exoskeleton, but not in mammals. Diflubenzuron was approved and is currently used for the control of a range of plagues in agriculture and parasites in livestock (5). However, toxicity studies have demonstrated potential side-effects in non-target animal models. Thus, the safety of these chemicals needs to be reassessed over time, particularly when new analytical methods or experimental models become available. Our hypothesis was that, within the current recommended doses, diflubenzuron treatment would not affect gamete or embryo quality and developmental potential in cattle, as determined by CASA or OPU-IVEP. The present study provided additional evidence of the lack of reproductive toxicity of diflubenzuron on livestock.

Although the focus of the current study was the potential effects on gametes and embryos, we also monitored weight and hematological parameters that could indicate systemic toxicity. We evaluated indicators of the hematopoietic system (hematocrit, protein), hepatic (alkaline phosphatase), and renal (creatinine) function. The liver is the main organ responsible for the metabolism of endogenous and exogenous compounds and, together with the kidney, is responsible for the excretion of most metabolites. Therefore, these organs are targets for the toxic actions of chemicals, resulting in altered plasma concentrations of biomarkers such as ALP and creatinine (23, 24). Moreover, ALT was found to have increased in male rats treated with Diflubenzuron (11). The hematopoietic system, on the other hand, is highly susceptible to toxic substances, being an important endpoint in toxicological studies (25). In the current study, no physiological endpoint was affected by the treatment with diflubenzuron. As expected, we observed fluctuations over time, all but for creatinine within the physiological range for cattle (26, 27). In both groups, creatinine plasma concentrations were consistently higher than the reference values used by the laboratory that performed the analyses. However, all animals remained sound and showed no other signs of potential intoxication throughout the experiment. One can speculate whether the higher creatinine concentrations observed were due to any of the environmental effects that may affect thresholds and the width of reference intervals, as reported elsewhere (27). There was also no effect of DIF treatment on body weight, which remained constant in heifers receiving a maintenance diet, whereas increased over time in bulls raised on pasture.

Fertility is a key component of toxicity tests. A number of chemicals have been described as endocrine disruptors for cattle (28), and thus with the potential to affect the reproductive process and to compromise fertility (29). However, the reproductive toxicity of diflubenzuron was evaluated mostly in non-target species, especially in rodents. de Barros et al. (11) observed that subacute exposure of male rats to diflubenzuron was associated with a decrease on testicle weight and daily sperm production, although no alterations were found in sperm morphology. The extrapolation of these results to other species, however, requires caution, particularly due to the differences in the physiology of both species and in the doses used. Interestingly, there are very few reports evaluating diflubenzuron toxicity on cattle reproduction. A study conducted in the late 1970′s with young Holstein male calves found no effect of diflubenzuron on sperm (30). However, in that study a single semen evaluation was performed. In the current study, the time-window (56 days) used almost encompassed the seminiferous epithelium cycle and the duration of spermatogenesis in cattle (31), so we were able to evaluate potential effects over time. Moreover, by using CASA, a much more sensitive analytic technique, we evaluated a range of sperm kinetic endpoints. Nevertheless, no evidence of detrimental effects of diflubenzuron was detected.

One potential bias of studies evaluating COC recovery and thus embryo yield is the uneven distribution of cows showing high or low AFC. Ovarian follicle population has a Weibull-like, rather than a Gaussian distribution, with a few animals presenting over 100 antral follicles at a given time (32). Donors yielding over 500 COC have been reported (33), thus a random distribution of donors within treatments may result in significant bias. In the current study, we used a balanced distribution strategy, ensuring that there were differences neither in AFC nor in the number of follicles aspirated or COC recovered at the first OPU-IVEP session between CG and DIF groups.

In heifers, as observed for bulls, there was no effect of diflubenzuron on any of the endpoints evaluated. Differences over time were expected, particularly due to the trend of reduction in COC recovery in *Bos taurus indicus* donors undergoing repeated OPU (34). To our best knowledge, this is the first study to address the potential hazard effects of diflubenzuron on oocyte quality and developmental potential in cattle. In insects, diflubenzuron acts not only on chitin formation, but also directly on reproduction in adult females, reducing follicle populations, yolk formation, and fecundity (13, 35). The mechanisms underlying the latter effects, however, are not fully understood. Potential detrimental effects on natural reproduction in large, mono-ovulating species such as cattle may be difficult to demonstrate, due to the high number of animals required and to the difficulty in controlling other environmental effects. In this regard, the OPU-IVEP model used was particularly important to ensure the evaluation of a high number of gametes (>800 per group), as well as the evaluation of their developmental potential during the critical period encompassing maturation, fertilization, and early embryo development. In both groups (CG and DIF), oocyte recovery and quality, as well as cleavage and embryo rates, were similar, and within those reported for the Nelore breed (20, 32, 36). Thus, the current results suggest that there is no interference of diflubenzuron treatment on the latter stages of folliculogenesis and oocyte maturation, differently to what is observed in non-chordate animals.

## 5. Conclusion

In summary, there is no evidence of a detrimental effect of short-term treatments with diflubenzuron, within the recommended dose, on gamete quality or embryo development potential in cattle.

## Data availability statement

The raw data supporting the conclusions of this article will be made available by the authors, without undue reservation.

## Ethics statement

The animal study was reviewed and approved by Embrapa Ethics in the Use of Animals Committee (Protocol CEUA-002/2022). Written informed consent was obtained from the owners for the participation of their animals in this study.

## Author contributions

MP and JV: conceptualization, supervision, and methodology. MP, RF, and JV: funding. MX, LM, RM, DM, JB, and JV: investigation. JV: statistical analysis. MX, RF, RA, and JV: writing—original draft and writing—review and editing. All authors contributed to the article and approved the submitted version.

## References

[1] Sun R Liu C Zhang H Wang Q. Benzoylurea chitin synthesis inhibitors. J Agric Food Chem. (2015) 63:6847–65. 10.1021/acs.jafc.5b02460 26168369

[2] Ivie GW. Fate of diflubenzuron in cattle and sheep. J Agric Food Chem. (1978) 26:81–9. 10.1021/jf60215a013 340489

[3] Tfouni SAV Furlani RPZ Carreiro LB Loredo ISD Gomes AG Alves LA. Determination of diflubenzuron residues in milk and cattle tissues. Arq Br Med Vet Zoot. (2013) 65:301–7. 10.1590/S0102-09352013000100043

[4] FAO. (2011). Available online at: https://www.fao.org/fileadmin/templates/agphome/documents/Pests_Pesticides/JMPR/Report11/Diflubenzuron.pdf (accessed May 2, 2023).

[5] Junquera P Hosking B Gameiro M Macdonald A. Benzoylphenyl ureas as veterinary antiparasitics. An overview and outlook with emphasis on efficacy, usage and resistance. Parasite. (2019) 26:26. 10.1051/parasite/2019026 31041897 PMC6492539

[6] Van der Leek ML Donovan GA Saltman RL Moja RJ. Effect of an insecticide controlled-release bolus on a milk antibiotic residue test. J Dairy Sci. (1991) 74:433–5. 10.3168/jds.S0022-0302(91)78188-5 2045550

[7] Perocco P Colacci A Grilli S. In vitro cytotoxic and cell transforming activities exerted by the pesticides cyanazine, dithianon, diflubenzuron, procymidone, and vinclozolin on BALB/c 3T3 cells. Environ Mol Mutagen. (1993) 21:81–6. 10.1002/em.2850210111 8419158

[8] Bayoumi AE Pérez-Pertejo Y Zidan HZ Balaña-Fouce R Ordóñez C Ordóñez D. Cytotoxic effects of two antimolting insecticides in mammalian CHO-K1 cells. Ecotoxicol Environ Saf. (2003) 55:19–23. 10.1016/S0147-6513(02)00068-4 12706389

[9] de Barros AL de Souza VV Navarro SD Oesterreich SA Oliveira RJ Kassuya CA. Genotoxic and mutagenic effects of diflubenzuron, an insect growth regulator, on mice. J Toxicol Environ Health A. (2013) 76:1003–6. 10.1080/15287394.2013.830585 24168035

[10] Quarles JM Norman JO Kubena LF. Absence of transformation by diflubenzuron in a host-mediated transplacental carcinogen assay. Bull Environ Contam Toxicol. (1980) 25:252–6. 10.1007/BF01985520 6775716

[11] de Barros AL Cavalheiro GF de Souza AV Traesel GK Anselmo-Franci JA Kassuya CA. Subacute toxicity assessment of diflubenzuron, an insect growth regulator, in adult male rats. Environ Toxicol. (2016) 31:407–14. 10.1002/tox.22054 25266294

[12] Lee W An G Park H Lim W Song G. Diflubenzuron leads to apoptotic cell death through ROS generation and mitochondrial dysfunction in bovine mammary epithelial cells. Pestic Biochem Physiol. (2021) 177:104893. 10.1016/j.pestbp.2021.104893 34301355

[13] Smagghe G Zotti M Retnakaran A. Targeting female reproduction in insects with biorational insecticides for pest management: a critical review with suggestions for future research. Curr Opin Insect Sci. (2019) 31:65–9. 10.1016/j.cois.2018.10.009 31109675

[14] Viana J. 2020 Statistics of embryo collection and transfer in domestic farm animals: world embryo industry grows despite the pandemic. Embr Tech Newsl. (2021) 39:24–37.

[15] Luciano AM Franciosi F Lodde V Corbani D Lazzari G Crotti G. Transferability and inter-laboratory variability assessment of the in vitro bovine oocyte maturation (IVM) test within *ReProTect*. *Reprod Toxicol*. (2010) 30:81–8. 10.1016/j.reprotox.2010.01.015 20156549

[16] Santos RR Schoevers EJ Roelen BA. Usefulness of bovine and porcine IVM/IVF models for reproductive toxicology. Reprod Biol Endocrinol. (2014) 12:117. 10.1186/1477-7827-12-117 25427762 PMC4258035

[17] Evans AC Mossa F Fair T Lonergan P Butler ST Zielak-Steciwko AE. Causes and consequences of the variation in the number of ovarian follicles in cattle. Soc Reprod Fertil Suppl. (2010) 67:421–9. 10.7313/UPO9781907284991.032 21755688

[18] Doroteu EM Viana JHM Ferreira Junior JA Macedo JTA Oliveira RA Pedroso PMO. Effect of a single or two doses of an anti-GnRH vaccine on testicle morpho-functional characteristics in Nelore bulls. Trop Anim Health Prod. (2021) 53:153. 10.1007/s11250-021-02600-x 33547980 PMC7867542

[19] Menon AG Barkema HW Wilde R Kastelic JP Thundathil JC. Associations between sperm abnormalities, breed, age, and scrotal circumference in beef bulls. Can J Vet Res. (2011) 75:241–7. 22468020 PMC3187629

[20] Malard PF Peixer MAS Grazia JG Brunel HDSS Feres LF Villarroel CL. Intraovarian injection of mesenchymal stem cells improves oocyte yield and in vitro embryo production in a bovine model of fertility loss. Sci Rep. (2020) 10:8018. 10.1038/s41598-020-64810-x 32415089 PMC7229041

[21] Viana JH De Almeida Camargo LS De Moraes Ferreira A De Sa WF De Carvalho Fernandes CA De Pinho Marques Junior A. Short intervals between ultrasonographically guided follicle aspiration improve oocyte quality but do not prevent establishment of dominant follicles in the Gir breed (Bos indicus) of cattle. Anim Reprod Sci. (2004) 84:1–12. 10.1016/j.anireprosci.2003.12.002 15302383

[22] Johnson WH. The significance to bull fertility of morphologically abnormal sperm. Vet Clin North Am Food Anim Pract. (1997) 13:255–70. 10.1016/S0749-0720(15)30339-X 9216047

[23] Stark JL. BUN/creatinine: your keys to kidney function. Nursing. (1980) 10:33–8. 10.1097/00152193-198005000-00007 6899894

[24] Ozer J Ratner M Shaw M Bailey W Schomaker S. The current state of serum biomarkers of hepatotoxicity. Toxicology. (2008) 245:194–205. 10.1016/j.tox.2007.11.021 18291570

[25] Gribaldo L. Haematotoxicology: scientific basis and regulatory aspects. Altern Lab Anim. (2002) 30 Suppl 2:111–3. 10.1177/026119290203002S17 12513660

[26] Rowlands GJ A. review of variations in the concentrations of metabolites in the blood of beef and dairy cattle associated with physiology, nutrition and disease, with particular reference to the interpretation of metabolic profiles. World Rev Nutr Diet. (1980) 35:172–235. 10.1159/000386410 6994374

[27] Roland L Drillich M Iwersen M. Hematology as a diagnostic tool in bovine medicine. J Vet Diagn Invest. (2014) 26:592–8. 10.1177/1040638714546490 25121728

[28] Petro EM Leroy JL Van Cruchten SJ Covaci A Jorssen EP Bols PE. Endocrine disruptors and female fertility: focus on (bovine) ovarian follicular physiology. Theriogenology. (2012) 78:1887–900. 10.1016/j.theriogenology.2012.06.011 22925646

[29] Mourikes VE Flaws JA. Reproductive toxicology: effects of chemical mixtures on the ovary. Reproduction. (2021) 162:F91–F100. 10.1530/REP-20-0587 33528380 PMC8325701

[30] Miller RW Cecil HC Carey AM Corley C Kiddy CA. Effects of feeding diflubenzuron to young male holstein cattle. Bull Environ Contam Toxicol. (1979) 23:482–6. 10.1007/BF01769991 387118

[31] Staub C Johnson L. Review: spermatogenesis in the bull. Animal. (2018) 12:s27–35. 10.1017/S1751731118000435 29882505

[32] Pontes JH Melo Sterza FA Basso AC Ferreira CR Sanches BV Rubin KC. Ovum pick up, in vitro embryo production, and pregnancy rates from a large-scale commercial program using Nelore cattle (Bos indicus) donors. Theriogenology. (2011) 75:1640–6. 10.1016/j.theriogenology.2010.12.026 21334055

[33] Resende AO Bohrer RC Viana JHM. A record of 485 viable cumulus-oocyte complexes recovered and 165 viable embryos produced in a single ovum pickup session from a Senepol breed donor. Reprod Fertil Develop. (2021) 33:111. 10.1071/RDv33n2Ab7

[34] Gimenes LU Ferraz ML Fantinato-Neto P Chiaratti MR Mesquita LG Sá Filho MF. The interval between the emergence of pharmacologically synchronized ovarian follicular waves and ovum pickup does not significantly affect in vitro embryo production in Bos indicus, Bos taurus, and Bubalus bubalis. Theriogenology. (2015) 83:385–93. 10.1016/j.theriogenology.2014.09.030 25447149

[35] Soltani-Mazouni N. Effects of ingested diflubenzuron on ovarian development during the sexual maturation of mealworms. Tissue Cell. (1994) 26:439–45. 10.1016/0040-8166(94)90027-2 18621275

[36] Pontes JH Nonato-Junior I Sanches BV Ereno-Junior JC Uvo S Barreiros TR. Comparison of embryo yield and pregnancy rate between in vivo and in vitro methods in the same Nelore (Bos indicus) donor cows. Theriogenology. (2009) 71:690–7. 10.1016/j.theriogenology.2008.09.031 18995895

